# Evidence that a transcription factor regulatory network coordinates oxidative stress response and secondary metabolism in aspergilli

**DOI:** 10.1002/mbo3.63

**Published:** 2013-01-01

**Authors:** Sung-Yong Hong, Ludmila V Roze, Josephine Wee, John E Linz

**Affiliations:** 1 Department of Food Science and Human Nutrition, Michigan State UniversityEast Lansing, Michigan, 48824; 2 Department of Microbiology and Molecular Genetics, Michigan State UniversityEast Lansing, Michigan, 48824; 3 National Food Safety and Toxicology Center, Michigan State UniversityEast Lansing, Michigan, 48824; 4 Center for Integrative Toxicology, Michigan State UniversityEast Lansing, Michigan, 48824

**Keywords:** Aflatoxin, *Aspergillus parasiticus*, oxidative stress, reactive oxygen species, secondary metabolism

## Abstract

The mycotoxin aflatoxin is a secondary metabolite and potent human carcinogen. We investigated one mechanism that links stress response with coordinate activation of genes involved in aflatoxin biosynthesis in *Aspergillus parasiticus*. Electrophoretic mobility shift assays demonstrated that AtfB, a basic leucine zipper (bZIP) transcription factor, is a master co-regulator that binds promoters of early (*fas-1*), middle (*ver-1*), and late (*omtA*) aflatoxin biosynthetic genes as well as stress-response genes (mycelia-specific *cat1* and mitochondria-specific Mn *sod*) at cAMP response element motifs. A novel conserved motif 5′-T/GNT/CAAG CCNNG/AA/GC/ANT/C-3′ was identified in promoters of the aflatoxin biosynthetic and stress-response genes. A search for transcription factors identified SrrA as a transcription factor that could bind to the motif. Moreover, we also identified a STRE motif (5′-CCCCT-3′) in promoters of aflatoxin biosynthetic and stress-response genes, and competition EMSA suggested that MsnA binds to this motif. Our study for the first time provides strong evidence to suggest that at least four transcription factors (AtfB, SrrA, AP-1, and MsnA) participate in a regulatory network that induces aflatoxin biosynthesis as part of the cellular response to oxidative stress in *A. parasiticus*.

## Introduction

Reactive oxygen species such as singlet oxygen (^1^O_2_), superoxide (O_2_·^−^), hydrogen peroxide (H_2_O_2_), and hydroxyl radical (HO·) cause oxidative damage to protein, DNA, and lipids through protein inactivation and lipid peroxidation in cells. Reactive oxygen species (ROS) is produced either by exposure to environmental agents such as UV and nutrient starvation or endogenously as by-products of metabolic pathways in different cellular compartments (e.g., respiration in mitochondria and β-oxidation of fatty acids in peroxisomes) (Apel and Hirt 2004). To cope with increased ROS levels, cells evolved various means to detoxify ROS including antioxidant enzymes (e.g., catalases, peroxidases, and superoxide dismutase), and glutathione and thioredoxin systems (Apel and Hirt 2004; Gessler et al. 2007). Of these, two major antioxidant enzymes, superoxide dismutase (SOD) and catalase, play important roles in the ROS-scavenging mechanism. SOD catalyzes dismutation of superoxide to H_2_O_2_ and catalase detoxifies H_2_O_2_ to H_2_O and O_2_. As part of the defense mechanism of cells, ROS was proposed to act as a second messenger to activate antioxidant gene transcription in response to oxidative stress and to regulate development and differentiation in molds, budding, and fission yeasts, as well as plants (Toone and Jones 1998; Apel and Hirt 2004; Miskei et al. 2009).

In budding and fission yeasts, histidine kinases in two-component signaling systems function as sensors of oxidative stress and activate mitogen-activated protein kinase (MAPK)-signaling cascades, resulting in induction of defense gene expression (Bahn et al. 2007). In the fission yeast *Schizosaccharomyces pombe*, the basic leucine zipper (bZIP) transcription factor Atf1, a member of the ATF/cAMP response element binding (CREB) protein DNA-binding family, is activated by the stress-activated MAPK Sty1/Spc1 through a MAPK cascade. Atf1 then positively regulates entry of the cell into stationary growth phase (Moye-Rowley 2002). Expression of the cytosolic catalase T gene, *ctt1*, is strongly dependent on Atf1 and another bZIP-type transcription factor, Pap1, in *S. pombe* in response to oxidative stress (Nguyen et al. 2000; Moye-Rowley 2002). In the budding yeast *Saccharomyces cerevisiae*, Sko1 (a homolog of Atf1 in *S. pombe*) binds to a cAMP-response element consensus sequence (5′-TG/TACGTC/AA-3′) in promoters of stress-response genes (Proft et al. 2005).

Filamentous fungi use similar stress-response signaling pathways to those in yeast (Kawasaki et al. 2002; Furukawa et al. 2005; Hagiwara et al. 2007; Lara-Rojas et al. 2011). Homologs of almost all components of the stress-activated protein kinase (SAPK)-signaling cascade in yeast were also found in *Aspergillus nidulans* (Miskei et al. 2009). For example, AtfA, a homolog of Atf1 in *S. pombe*, plays an important role in tolerance of conidia to oxidative stress in *A. nidulans* while NapA, a homolog of Pap1 in *S. pombe*, is responsible for hyphal tolerance to oxidative stress (Asano et al. 2007; Hagiwara et al. 2008). In *Aspergillus oryzae*, *atfA* and *atfB* were identified and *atfA* was expressed throughout growth while *atfB* was expressed late in the growth phase and mediated oxidative stress tolerance in conidia generated on a solid growth medium (Sakamoto et al. 2008). The authors showed that AtfB positively regulates target genes including conidia-specific *catA*, and these genes carry CRE motifs (5′-TGACGTCA-3′) in their promoters. Specific AtfB binding to these promoters was not demonstrated.

*Aspergillus parasiticus* and *Aspergillus flavus* are two major producers of aflatoxin which is closely associated with human liver cancer. Others reported a correlation between ROS formation, aflatoxin production, and antioxidant enzyme activation (Fanelli et al. 1984; Narasaiah et al. 2006; Reverberi et al. 2006). Aflatoxin production in *A. parasiticus* was increased by oxidative stress (Jayashree and Subramanyam 2000), and it was reduced by antioxidants including eugenol, by stimulation of antioxidant enzyme activities such as catalase and SOD, or by ROS scavengers including ethylene (Jayashree and Subramanyam 1999; Reverberi et al. 2005; Huang et al. 2009). Surprisingly, a toxigenic strain of *A. parasiticus* showed increased activities of antioxidant enzymes such as SOD compared with the nontoxigenic strain and disruption of an SOD gene in *A. flavus* resulted in decreased aflatoxin production (Narasaiah et al. 2006; He et al. 2007). In *A. parasiticus*, disruption of Ap*yapA*, an ortholog of *yap1* in *S. cerevisiae*, resulted in precocious and increased ROS formation and precocious aflatoxin production (Reverberi et al. 2007, 2008).

Taken together, the evidence strongly suggests that aflatoxin biosynthesis is part of the fungal response to oxidative stress. The mechanisms that activate transcription of genes involved in aflatoxin biosynthesis (for clarity, we will designate these as aflatoxin biosynthetic genes or aflatoxin genes) in response to oxidative stress are not fully understood. However, our recent studies shed light on the regulatory mechanisms that integrate secondary metabolism and cellular response to oxidative stress (Roze et al. 2004, 2011). In these previous studies, we demonstrated that AtfB binds to promoters of seven aflatoxin genes which carry CRE motifs using chromatin immunoprecipitation (ChIP) in *A. parasiticus* grown under aflatoxin-inducing conditions (Roze et al. 2011). Electrophoretic mobility shift assays (EMSA) also showed that AtfB binds to CRE1 (5′-TGACATAA-3′) and AP-1 (5′-TGAGTAC-3′) binding sites in the promoter of *nor-1*, an early aflatoxin gene.

In this study, we investigated the mechanism of transcriptional co-regulation of antioxidant genes (mycelia-specific *cat1* and mitochondria-specific Mn *sod*) and secondary metabolism genes (the aflatoxin biosynthetic genes *fas-1*, *ver-1*, and *omtA* representing early, middle, and late aflatoxin biosynthetic genes, respectively) as a mean of cellular response to oxidative stress in *A. parasiticus*. We demonstrate using EMSA that the stress-related transcription factor AtfB binds to the promoters of early, middle, and late aflatoxin biosynthetic genes as well as stress-response genes that carry at least one CRE motif, under aflatoxin-inducing conditions, but AtfB does not bind to the *vbs* promoter, which lacks a CRE site under the same growth condition. Based on nucleotide sequence and EMSA analyses, we also propose that three other transcription factors, MsnA, SrrA, and AP-1, participate in transcriptional co-regulation of stress-response and aflatoxin biosynthetic genes. Furthermore, we found a conserved motif 5′-T/GNT/CAAGCCNNG/AA/GC/ANT/C-3′ in the promoters of *fas-1*, *ver-1*, *nor-1*, mycelial *cat1*, and Mn *sod*, which extends the conserved 8-base motif 5′-AGCCG/CTG/CA/G-3′ identified in the promoter of the *nor-1* in our previous study; the core consensus sequence 5′-AAGCC-3′ in the conserved motif is a sequence-specific-binding motif for the transcription factor SrrA. We also found a motif (5′-CCCCT-3′) for a stress-response element (STRE) in the promoters of the aflatoxin biosynthetic genes, *fas-1* and *ver-1,* as well as antioxidant genes, mycelial *cat1* and Mn *sod*. Competition EMSA analysis suggests that MsnA, which binds to STREs, is one player in transcriptional co-regulation. These data contribute to our understanding of the molecular mechanisms of transcriptional activation of aflatoxin biosynthetic genes and stress-response genes in response to oxidative stress in *A. parasiticus*.

## Experimental Procedures

### Strains, growth media, and growth conditions

*Aspergillus parasiticus* SU-1 (ATCC 56775) was used as a wild-type strain for aflatoxin production in this study. YES liquid medium (2% yeast extract, 6% sucrose; pH 5.8) was used as an aflatoxin-inducing medium. A 100 mL of YES medium in a 250-mL flask with five 6-mm glass beads was inoculated with 1 × 10^4^ spores mL^−1^ and incubated at 30°C in the dark with shaking at 150 rpm as described previously (Roze et al. 2011).

### Generation of anti-AP-1 antibodies

#### Design of peptide antigens

A BLAST search of the *A. flavus* genome database (http://www.aspergillusflavus.org/genomics) using a partial sequence of *A. parasiticus AP-1* (Reverberi et al. 2008) identified a single gene AFLA_129340 corresponding to a putative bZIP transcription factor, AP-1. Peptide antigens KQQNDKQRSQAKTDPD (amino acids 34–49; peptide 102) and VNQKDVEDIMGRVK (amino acids 571–584; peptide 103) were designed using Antigen Profiler™ (Thermo Fischer Scientific, Open Biosystems, Huntsville, AL).

#### Immunization of rabbits

Synthetic peptides 102 and 103 carrying Cys on the C terminus were conjugated to the carrier protein keyhole limpet hemocyanin and injected into New Zealand White rabbits as described previously (Roze et al. 2011). Specificity and titer of antisera were determined for each peptide using ELISA.

#### Immunoaffinity antibody purification

Polyclonal antibodies were purified from crude antiserum using an affinity column containing synthetic peptide coupled to Sepharose beads through an N-terminal cystein residue. Antibodies bound to the column were eluted into neutralizing 1 mol/L Tris buffer using a stepwise pH gradient. Purified antibodies were stored in antimicrobial saline (pH 8.2) at +4°C or −80°C.

### Western blot analysis

*Aspergillus parasiticus* SU-1 was grown in YES medium for designated periods of time and mycelia were harvested by filtration through Miracloth. Mycelia were frozen in liquid nitrogen. Frozen mycelia were ground in liquid nitrogen with a mortar and pestle, and resuspended at a 1:1 (w/v) ratio in TSA buffer (0.01 mol/L Tris, 0.15 mol/L NaCl, 0.05% NaN_3_, pH 8.0) containing one tablet of Complete mini protease inhibitor mixture (Roche Diagnostics, Indianapolis, IN), 50 μL of proteinase inhibitor mixture (Sigma-Aldrich, St. Louis, MO), and 0.125 mmol/L phenylmethylsulfonyl fluoride per 10 mL. Total protein extracts (90 μg per lane) or enriched nuclear protein extracts (200 μg per lane) were separated by electrophoresis on 12% SDS-polyacrylamide gels, transferred onto polyvinylidene difluoride (PVDF) membranes (PerkinElmer Life Sciences, Waltham, MA), and probed with anti-AP-1 antibodies (102 or 103; 5 μg mL^−1^) as a primary antibody. The membranes were incubated with goat anti-rabbit secondary antibody conjugated to the fluorescent tag IRDye 680 (Li-Cor Biosciences, Lincoln, NE). Protein bands were detected using an Odyssey infrared imaging system (Li-Cor Biosciences) at 795 nm.

### Preparation of cell extracts enriched in nuclear proteins for electrophoretic mobility shift assays

Preparation of cell extracts enriched for nuclear proteins from *A. parasiticus* SU-1 for EMSA was performed as described previously (Roze et al. 2004). Briefly, 100 mL of YES medium was inoculated with conidiospores (1 × 10^4^ mL^−1^) and incubated for 24, 48, or 60 h at 30°C with shaking at 150 rpm. The mycelia were harvested by filtration through Miracloth, ground in liquid nitrogen using a mortar and pestle, and resuspended in lysis buffer as described previously. The proteins were precipitated by ammonium sulfate (10% and then 70%), pelleted by centrifugation, resuspended in dialysis buffer, and dialyzed against the dialysis buffer. The dialyzed protein solution was aliquated and stored at −80°C.

### Electrophoretic mobility shift assays

**Figure 1:**
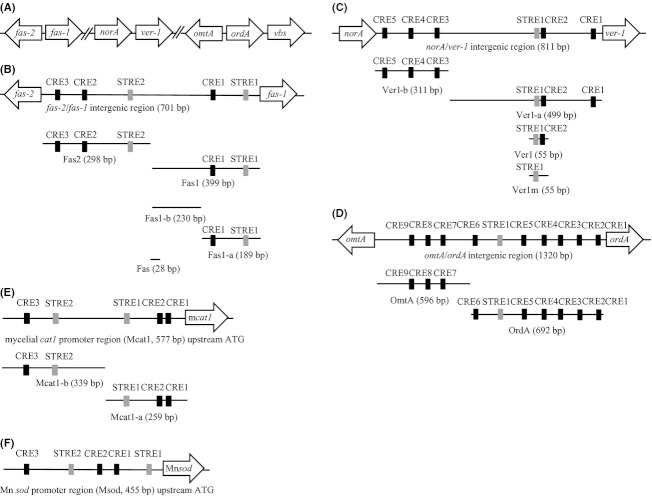
Promoter regions of aflatoxin biosynthetic and antioxidant genes used in electrophoretic mobility shift assays. (A) Schematic representation of the relevant genes in the aflatoxin gene cluster. (B) Schematic of *fas-2*/*fas-1* intergenic region. The *fas-2*/*fas-1* intergenic region was divided into two fragments designated Fas2 and Fas1. The Fas1 fragment was further subdivided into two smaller fragments designated Fas1-b and Fas1-a. Five putative *cis*-acting sites are shown including CRE and stress-response element (STRE) sites. The position of a 28-bp Fas competitor is shown. (C) Schematic of *norA*/*ver-1* intergenic region. The *norA*/*ver-1* intergenic region was divided into two fragments designated Ver1-b and Ver1-a. Six putative *cis*-acting sites are shown including CRE and STRE sites. The position of 55-bp Ver1 and Ver1 m competitors is shown. (D) Schematic of *omtA*/*ordA* intergenic region. The *omtA*/*ordA* intergenic region was divided into two fragments designated OmtA and OrdA. Ten putative *cis*-acting sites are shown including CRE and STRE sites. (E) Schematic of mycelial *cat1* promoter region designated Mcat1. The mycelial *cat1* promoter region was divided into two fragments designated Mcat1-b and Mcat1-a. Five putative *cis*-acting sites are shown including CRE and STRE sites. (F) Schematic of Mn *sod* promoter region designated Msod. Five putative *cis*-acting sites are shown including CRE and STRE sites.

Electrophoretic mobility shift assays, competition EMSA, and shift inhibition EMSA were performed as described previously (Roze et al. 2004). Double-stranded DNA fragments derived from the promoter region of *fas-1*, *ver-1*, *omtA*, *vbs*, mycelial *cat1*, and Mn *sod* were generated by PCR and gel purified using a Wizard SV gel and PCR cleanup system (Promega, Madison, WI). Primers used to generate PCR fragments are shown in Table S1. The DNA fragments were 5′ end-labeled with [γ-^32^P]ATP using USB OptiKinase (Affymetrix, Cleveland, OH), purified using a Micro Bio-Spin P-30 chromatography column (Bio-rad, Hercules, CA), and used as probes. Two alternative methods were used to generate double-stranded DNA competitor fragments: (1) A 51 bp NorR4 fragment was generated by PCR using pUC19GUS as a template and gel purified using a Wizard SV gel and PCR cleanup system (Promega) (Roze et al. 2011). Primers used to generate NorR4 are shown in Table S1. (2) A 28-bp Fas fragment including the motif 5′-AGCCGTGA-3′ (Roze et al. 2011) derived from the *fas2/fas1* intergenic region was generated by annealing of two complementary single-stranded synthetic oligonucleotides (Fig. 1B). The sequences used to generate the Fas fragment are shown in Table S1. This same strategy was used to generate a 55-bp Ver1 fragment, which carried two motifs (STRE1: CCCCT and CRE2: TGACCCAG), and a 55-bp Ver1 m fragment including the STRE motif only (Ver1 with CRE2 changed from TGAC to CTTC) (Fig. 1C) [Correction added on 04 February 2013, after first online publication: “CRE1” has been changed to “CRE2”.]. The sequences used to generate the Ver1 and Ver1 m fragments are shown in Table S1.

Electrophoresis on 5% nondenaturing polyacrylamide (80:1 acrylamide/bisacrylamide) gels was used to separate DNA–protein complexes. Twenty femtomole of each ^32^P-labeled probe was incubated for 15 min at 30°C with 2 μg of poly(dI-dC), 7.5 μg of bovine serum albumin, and competitor (if desired) with 5 μg of nuclear protein extract (added last) in a DNA-binding buffer (15% glycerol, 15 mmol/L HEPES [pH 7.9], 100 mmol/L KCl, 1 mmol/L EDTA, 2 mmol/L DTT) in 25 μL of final binding reaction. After separation, the gels were dried and exposed to x-ray film.

Polyclonal anti-AtfB antibodies (YSR) were used to block formation of DNA–protein complexes (shift inhibition EMSA) as described previously (Roze et al. 2011). Five micrograms of nuclear protein extracts were incubated in a DNA-binding buffer for 15 min at 30°C with 5 μL of anti-AtfB antibody (5.5 μg μL^−1^) or preimmune serum obtained from the same rabbit as the antibody was generated. Then, a ^32^P-labeled probe was added and the mixture was incubated for an additional 15 min at 30°C. Finally, DNA–protein complexes were resolved by electrophoresis.

In some reactions, rabbit polyclonal antibodies specific for AP-1 were used to block formation of DNA–protein complexes (shift inhibition EMSA) as described above.

### DNA sequence analysis of promoter regions in *A. parasiticus*

DNA sequence analysis of PCR products carrying promoter regions of *fas-1*, *ver-1*, *omtA*, *vbs*, mycelial *cat1*, and Mn *sod* was conducted at the Research Technology Support Facility (RTSF) at Michigan State University. Comparison of promoter sequences in *A. parasiticus* and *A*. *flavus* was performed using EMBOSS pairwise sequence alignment.

### Total RNA isolation and transcript level analysis by quantitative real-time PCR

Transcript levels in fungal cells were analyzed using real-time PCR as described previously (Roze et al. 2011). *Aspergillus parasiticus* SU-1 was cultured in YES liquid medium, and the mycelia were harvested and frozen in liquid N_2_ at 24, 48, or 60 h. Total RNA was isolated from two biological replicates by the TRIzol method (TRIzol Reagent; Invitrogen, Carlsbad, CA). Total RNA was treated with RNase-free DNase (Qiagen, Valencia, CA) and RNA quality was examined by an Agilent 2100 Bioanalyzer (Agilent Technologies, Santa Clara, CA). For reverse transcription, 1 μg of total RNA was treated with gDNA WipeOut, and cDNA was prepared with the QuantiTect reverse transcription kit (Qiagen). Primers were designed using Primer Express software 3.0 (Applied Biosystems, Carlsbad, CA) and PrimerQuest^SM^ (Integrated DNA Technologies, Coralville, IA). Primers used for quantitative real-time PCR (qRT-PCR) analysis are listed in Table S2. For qRT-PCR analysis, 25 ng of reverse-transcribed RNA was amplified using an ABI PRISM 7900HT sequence detection system with Power SYBR Green PCR Mastermix (Applied Biosystems). Reactions were carried out using an initial incubation of 2 min at 50°C and 10 min at 95°C. This was followed by 40 cycles consisting of 15 sec at 95°C (denaturation) and 1 min at 60°C (annealing and extension). Resulting data were analyzed by the relative quantification method using SDS 2.1 software (Applied Biosystems). Relative gene expression levels were determined by the standard curve method (Applied Biosystems). The standard curve was derived by plotting cycles to threshold values versus the logarithm of known concentration of reverse-transcribed RNA between the ranges of 0.001 and 100 ng per reaction. For each gene, the relative level of mRNA at each time point is represented by the fold change of mRNA levels of a target gene divided by mRNA levels of β-tubulin at the same time point. Statistical analysis for all qRT-PCR was performed using SigmaPlot® software (v 11.0; Systat Software Inc., San Jose, CA). qRT-PCR data are presented as mean ± SE, *n* = 4, and were analyzed by one-way repeated measure analysis of variance.

## Results

Preliminary EMSA studies detected DNA–protein complex formation between EMSA protein extracts from *A. parasiticus* grown in YES aflatoxin-inducing medium for 48 h and DNA fragments derived from the intergenic or promoter regions of the divergently transcribed *fas-2*/*fas-1* genes (the entire intergenic region, 701 bp), the *norA*/*ver-1* genes (the entire intergenic region, 811 bp), the divergently transcribed *omtA*/*ordA* genes (the entire intergenic region, 1320 bp), the mycelial *cat1* gene (577 bp upstream from ATG), and the Mn *sod* gene (455 bp upstream from ATG) (Fig. 1). In order to further characterize these DNA–protein interactions, these large DNA fragments were subdivided into smaller pieces. The *fas-2*/*fas-1* intergenic region was divided into Fas2 (298 bp) and Fas1 (399 bp). Fas1 was further split into Fas1-b (230 bp) and Fas1-a (189 bp). The *norA*/*ver-1* intergenic region was divided into Ver1-b (311 bp) and Ver1-a (499 bp). The *omtA*/*ordA* intergenic region was divided into OmtA (596 bp) and OrdA (692 bp). The Mcat1 fragment (577 bp upstream from ATG) was divided into Mcat1-b (339 bp) and Mcat1-a (259 bp) (Fig. 1). Fragments Fas1-a, Ver1-b, OmtA, Mcat1-a, and Msod demonstrated prominent DNA–protein complexes in EMSA, and therefore, we continued to employ those fragments in EMSA experiments described below.

### Time-course of DNA–protein complex formation in aflatoxin biosynthetic and stress-response gene promoters

**Figure 2:**
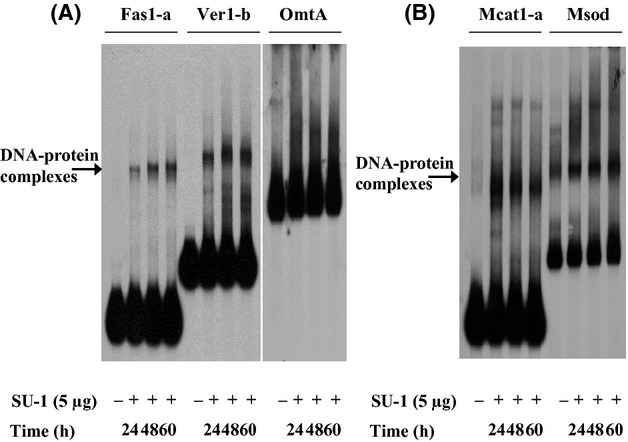
Time-course of DNA–protein complex formation as detected by electrophoretic mobility shift assays using aflatoxin (*fas-1* and *ver-1*) and antioxidant gene (mycelial *cat1* and Mn *sod*) promoters. *Aspergillus parasiticus* SU-1 was grown for 24, 48, or 60 h at 30°C in YES medium. Cell extracts enriched in nuclear proteins were prepared as described in Experimental Procedures. Five micrograms of enriched nuclear protein extracts was added to a P^32^-labeled promoter probes for each aflatoxin or antioxidant gene. (A) Fas1-a, Ver1-b, and OmtA probes. (B) Mcat1-a and Msod probes.

To correlate transcription factor-binding activity with activation of gene expression, EMSA was conducted on Fas1-a, Ver1-b, OmtA, Mcat1-a, and Msod promoter fragments using nuclear protein extracts from *A. parasiticus* grown in YES aflatoxin-inducing medium for 24 h (prior to aflatoxin gene expression), 48 h (during maximal rates of aflatoxin gene expression), or 60 h (after maximal rates of aflatoxin gene expression) (Trail et al. 1995). DNA–protein complexes were detected with Fas1-a, Ver1-b, and OmtA promoter fragments using the 24-h nuclear protein extract; similar sized but more abundant DNA–protein complexes and new complexes were observed with the same promoter fragments using 48- and 60-h nuclear protein extracts (Fig. 2A). These data are consistent with strong activation of the promoters of the aflatoxin biosynthetic genes at 48 and 60 h observed under aflatoxin-inducing conditions (Roze et al. 2011). The promoter of the stress-response gene, Mn *sod*, showed a similar pattern of DNA–protein complex formation as the aflatoxin biosynthetic genes (Fig. 2B). In contrast, for the promoter of the stress-response gene M*cat1* (Mcat1-a), the most abundant DNA–protein complexes were observed using the 24-h nuclear protein extract while DNA–protein complexes were observed at significantly reduced levels and some complexes disappeared using 48- or 60-h nuclear protein extracts (Fig. 2B). These data indicate that Mn *sod* and the aflatoxin genes *fas-1*, *ver-1*, and *omtA* are co-regulated while M*cat1* is differentially expressed early in growth.

### Identification of *cis*-acting-binding sites in the early and middle aflatoxin biosynthetic and stress-response gene promoters

**Figure 3:**
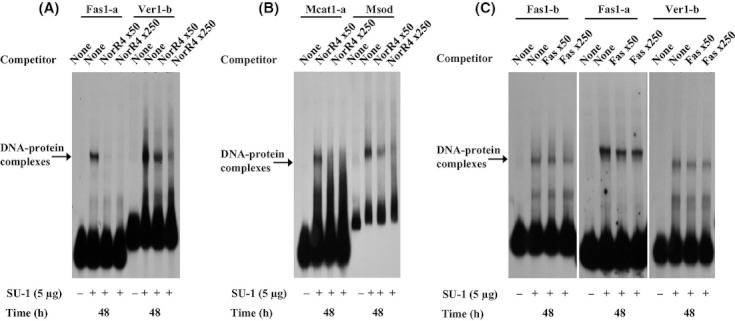
Competition electrophoretic mobility shift assays. *Aspergillus parasiticus* SU-1 was grown for 48 h at 30°C in YES medium. (A) Fas1-a and Ver1-b probes. (B) Mcat1-a and Msod probes. The 51-bp nonlabeled NorR4 fragment (contains CRE and AP-1 sites; 50- and 250-fold molar excess) was added to compete for labeled Fas1-a, Ver1-b, Mcat1-a, or Msod probes. (C) Fas1-b, Fas1-a, and Ver1-b probes. The 28-bp nonlabeled Fas fragment (contains 8-base motif, 5′-AGCCG/CTG/CA/G-3′; 50- and 250-fold molar excess) was added to compete for labeled Fas1-b, Fas1-a, or Ver1-b probes.

To locate specific *cis*-acting sites in promoters of *fas-1*, *ver-1*, mycelial *cat1*, and Mn *sod*, competition EMSA was performed. We previously demonstrated that a 51-bp NorR4 fragment derived from the *nor-1* promoter, contained CRE and AP-1 sites (5′-TGACATAA-3′ for CRE; and 5′-TGAGTAC-3′ for AP-1) and it competed efficiently for complex formation with a 170-bp NorR promoter fragment using a 48-h nuclear protein extract (Roze et al. 2011). We hypothesized that the promoters of other aflatoxin biosynthetic genes including *fas-1* and *ver-1* and the stress-response genes mycelial *cat1* and Mn *sod* are activated in response to oxidative stress in a similar way by binding identical transcription factors to analogous *cis*-acting elements in the promoters. Thus, first we used the 51-bp NorR4 fragment as a competitor in EMSA. The 51-bp NorR4 fragment containing both sites competed efficiently for DNA–protein complexes formed in Fas1-a, Ver1-b, Mcat1-a, and Msod promoter fragments using a 48-h nuclear protein extract (Fig. 3A and B). These data suggest that promoters of early (*fas-1*) and middle (*ver-1*) aflatoxin genes as well as stress-response genes (mycelial *cat1*, and Mn *sod*) carry similar CRE sites, AP-1 sites, or both sites as observed in the *nor-1* promoter.

We previously identified a conserved 8-base motif (5′-AGCCG/CTG/CA/G-3′) within promoters of eight aflatoxin genes by sequence-based MEME (a computer software package for sequence-based motif analysis of nucleotide sequences) motif analysis and showed AtfB binding to five of these eight promoters including *fas-1* and *nor-1* using ChIP analyses; the data suggested that the conserved 8-base motif may contribute to AtfB binding (Roze et al. 2011). Therefore, we tested whether the conserved 8-base motif actually binds AtfB or another transcription factor within the *fas-1* (which contains the motif) and *ver-1* (which does not contain the motif) promoters using competition EMSA. A 28-bp Fas fragment containing the 8-base motif, which was derived from the Fas1-b promoter fragment (Fig. 1B), competed weakly for DNA–protein complex formation in Fas1-b, Fas1-a, and Ver1-b promoter fragments using 48-h nuclear protein extract (Fig. 3C). The data suggest that the *ver-1* promoter carries a motif similar to the conserved 8-base motif in the *fas-1* promoter and that AtfB and/or another transcription factor binds specifically to this motif in the promoters of *fas-1* and *ver-1*.

### AtfB binds in aflatoxin biosynthetic and stress-response gene promoters that carry a CRE motif

Previously, we demonstrated that AtfB binds to the *nor-1* promoter using EMSA protein extracts from *A. parasiticus* grown for 48 h in YES aflatoxin-inducing medium and antibodies that specifically recognize AtfB (Roze et al. 2011). In this study, first, we determined whether AtfB binds early, middle, and late aflatoxin gene promoters that carry CRE sites (see Table 1) using nuclear protein extracts prepared from *A. parasiticus* grown in YES medium for 24, 48, or 60 h and AtfB-specific polyclonal antibodies to conduct shift inhibition EMSA.

**Table 1:** CRE sites in aflatoxin and antioxidant gene promoters used in this study

Aflatoxin and antioxidant gene promoters	Total number of all CRE sites containing TGAC	All CRE sites containing TGAC^1^
*fas-2/fas-1* (701 bp)	3	**TGACG**GAC, **TGAC**CGAT, **TGAC**AAGC
*norA*/*ver-1* (811 bp)	5	**TGACG**AG**A**, **TGAC**TGAG, **TGAC**CAAG, **TGAC**CCAG, **TGACG**GG**A**
*omtA*/*ordA* (1320 bp)	9	**TGAC**T**TC**G, **TGACG**CA**A**, **TGAC**TCGT, **TGAC**CAT**A**, **TGACG**C**CA**, **TGACG**AAC, **TGAC**CCAC, **TGACG**C**C**C, **TGACG**ATC
*ordA*/*vbs* (511 bp)	None	None
Mycelial *cat1* 577 bp upstream ATG	3	**TGAC**C**T**AC, **TGAC**ACTT, **TGAC**AGTT
Mn *sod* 455 bp upstream ATG	3	**TGAC**TGAC, **TGAC**TATG, **TGAC**CAG**A**

1 The nucleotides showing the same sequence with the consensus CRE motif (TGACGTCA) were indicated in bold.

**Figure 4:**
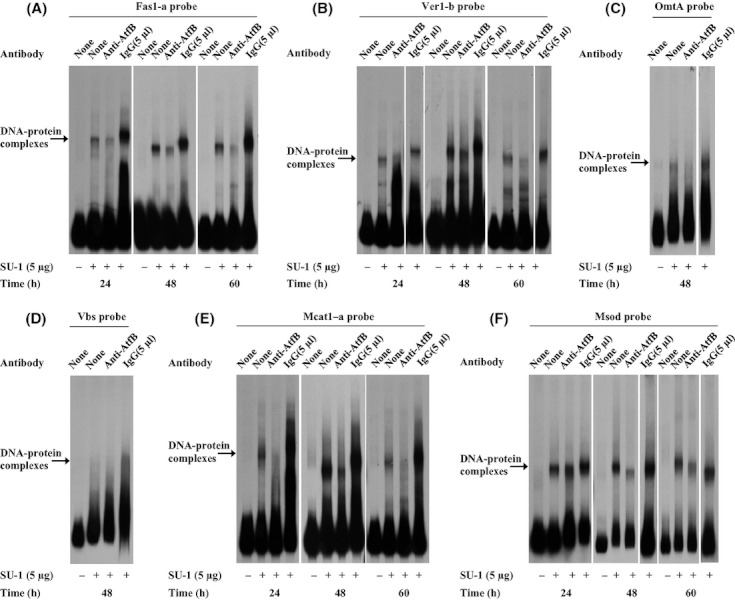
Electrophoretic mobility shift assays of AtfB binding in the *fas-1,* *ver-1,* *omtA,* *vbs*, mycelial *cat1*, and Mn *sod* promoters. *Aspergillus parasiticus* SU-1 was grown for 24, 48, or 60 h at 30°C in YES medium. Enriched nuclear protein extracts were prepared as described in Experimental Procedures. Five micrograms of enriched nuclear protein extracts was added to a P^32^-labeled promoter probe for each aflatoxin or antioxidant gene. Anti-AtfB antibodies (YSR) or preimmune serum was added to determine whether these could block protein/DNA interaction (shift inhibition). (A) Fas1-a probe. Nuclear protein extracts were used from 24-, 48-, or 60-h culture. (B) Ver1-b probe. Nuclear protein extracts were used from 24-, 48-, or 60-h culture. (C) OmtA probe. Nuclear protein extracts were used from 48-h culture. (D) Vbs probe. Nuclear protein extracts were used from 48-h culture. (E) Mcat1-a probe. Nuclear protein extracts were used from 24-, 48-, or 60-h culture. (F) Msod probe. Nuclear protein extracts were used from 24-, 48-, or 60-h culture.

DNA–protein complexes were observed with Fas1-a, Ver1-b, and OmtA promoter fragments using 24-, 48-, or 60-h nuclear protein extracts and complex formation was inhibited by pretreatment of the nuclear protein extracts with anti-AtfB antibodies before addition of the promoter fragment but not by preimmune serum obtained from the same rabbit that the antibody was generated (Fig. 4A–D). These data suggest that complex formation is due to specific AtfB binding to the promoter fragments at all time points analyzed. The Ver1-b probe produced two DNA–protein complexes (upper and lower) as shown in Figures 2A, 3C, and 4B. Only the upper complex contains AtfB, because the complex was competed by anti-AtfB (Fig. 4B); the nature of the protein(s) in the lower band is unknown. Similarly, the Fas1-b promoter fragment demonstrates two complexes as shown in Figure 3C. Only the upper complex with higher intensity competed by Fas-unlabeled probe. These findings are also consistent with previous results obtained with anti-AtfB antibodies in ChIP analysis that showed enrichment of AtfB binding to each promoter carrying CRE sites under aflatoxin-inducing conditions (Roze et al. 2011).

As a control, we used a *vbs* promoter fragment (Vbs: 508 bp), which lacks CRE sites (Table 1) (Roze et al. 2004), for EMSA. Vbs-protein complex formation was not observed using a 48-h nuclear protein extract (Fig. 4D). These data confirm previous results obtained using ChIP analyses, in which the *vbs* promoter did not bind AtfB at any time point under aflatoxin-inducing conditions (Roze et al. 2011).

When taken together, these results indicate that AtfB binds at early (fas-1), middle (ver-1), and late (omtA) aflatoxin biosynthetic gene promoters that carry CRE sites.

Next, to determine whether AtfB also binds at stress-response gene promoters that carry CRE sites (see Table 1), shift inhibition EMSA was performed on the mycelial *cat1* and Mn *sod* promoters. A Mcat1-a-protein complex was observed in EMSA using 24-, 48-, or 60-h nuclear protein extracts (Fig. 4E) and complex formation was inhibited by pretreatment of the nuclear protein extracts with anti-AtfB antibodies similar to results observed for the aflatoxin biosynthetic genes (Fig. 4E). For a second stress-response gene, an Mn *sod* promoter (Msod, 455 bp)–protein complex was detected by EMSA using a 24-, 48-, or 60-h nuclear protein extracts (Fig. 4F) and complex formation was inhibited by pretreatment of the nuclear protein extracts with anti-AtfB antibody using 48- or 60-h nuclear protein extracts. However, antibody pretreatment did not prevent complex formation when the 24-h protein extract was used (Fig. 4F). These results imply that the DNA–protein complex formation on the Mn *sod* promoter depends on AtfB at 48 and 60 h, but not at 24 h.

### AP-1-specific polyclonal antibodies do not block DNA–protein complex formation

As previous data suggested that an AP-1 site (5′-TGAGTAC-3′) played a role in activation of the *nor-1* promoter, we tested whether DNA–protein complex formation in other aflatoxin genes and stress-response genes is inhibited by pretreatment of the nuclear protein extract with AP-1-specific antibodies (shift inhibition EMSA). To demonstrate antibody specificity, we conducted Western blot analysis of the *A. parasiticus* nuclear protein extract. AP-1 antibodies detected a 64-kDa protein in the *A. parasiticus* nuclear protein extract (Fig. S1), and this mass agreed with the predicted molecular mass of AP-1 (63.8 kDa) in *A. flavus* (GenBank accession no: EED48711.1). AP-1 antibody pretreatment of the nuclear protein extracts did not affect DNA–protein complex formation within the Fas1-a, Ver-1b, Mcat1-a, or Msod promoter fragments (Fig. S2) suggesting that AP-1 does not bind directly to promoters of these target genes.

### Aflatoxin biosynthetic and stress-response gene promoters contain a conserved AAGCC motif

**Figure 5:**
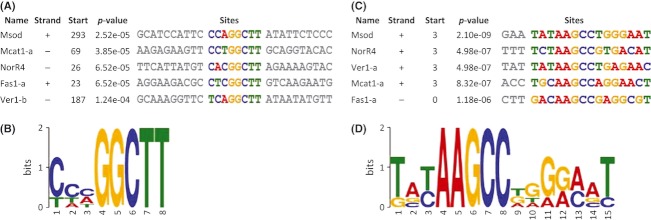
Sequence-based motif analysis of *fas-1,* *ver-1*, mycelial *cat1*, and Mn *sod* promoters using MEME. Fas1-a, Ver1-b, Mcat1-a, and Msod promoters fragment and the NorR4 competitor used for competition electrophoretic mobility shift assays were analyzed for conserved motifs using sequence-based MEME motif analysis. A conserved motif (5′-T/GNT/CAAGCCNNG/AA/GC/ANT/C-3′) was found in the promoter regions, where the core consensus sequence was 5′-AAGCC-3′. (A) Sites carrying the core consensus sequence in five promoter fragments. (B) Sequence logo of the core consensus sequence. (C) Sites carrying the conserved motif in five promoter fragments. (D) Sequence logo of the conserved motif.

Because shift inhibition EMSA analysis demonstrated that AtfB binds specifically to Fas1, Ver1, Mcat1, and Msod promoter fragments and that the NorR4 fragment competed efficiently for DNA–protein complexes formed in the promoter fragments, we applied sequence-based MEME motif analysis to search for motifs that were conserved in these promoters and in the NorR4 competitor fragment. A 5-base conserved motif 5′-AAGCC-3′ was found in all five promoter regions (Fig. 5A and B), and this motif is a part of the 8-base motif 5′-AGCCG/CTG/CA/G-3′ identified in the *nor-1* promoter in previous work (Roze et al. 2011). Sequence-based MEME analysis of these same five promoters extended the 8-base motif to a 15-base conserved motif 5′-T/GNT/CAAGCCNNG/AA/GC/ANT/C-3′ (Fig. 5C and D). Interestingly, the conserved core consensus sequence 5′-AAGCC-3′ has been shown previously to be recognized by the transcription factor SrrA, an *A. parasiticus* ortholog of *S. cerevisiae* Skn7. Skn7 recruits Yap1 to the promoters of yeast target genes and activates cooperatively their expression in response to oxidative stress (He and Fassler 2005; Mulford and Fassler 2011); transcriptional activation occurs through sequence-specific interaction of Skn7 with one of four *cis*-regulatory elements within promoters (5′-GGCNGGC-3′, 5′-GGCNNGGC-3′, 5′-GGCNAGA-3′, or 5′-GGCNNAGA-3′). We observed that the AP-1 and SrrA motifs are located in close proximity to each other, lying within 60 bp in the promoters of aflatoxin (*fas-1* and *ver-1*) and stress-response genes (mycelial *cat1* and Mn *sod*) (Table 2). Our findings are consistent with the observation that Yap1 and Skn7 recognition sites in *S. cerevisiae* are located within 20–50 bp of each other (He and Fassler 2005).

**Table 2:** AP-1 sites in aflatoxin and antioxidant gene promoters used in this study

Aflatoxin and antioxidant gene promoters	Total number of all AP-1 sites within 60 bp near SrrA site	All AP-1 sites within 60 bp near SrrA site^1^
Fas1-a (189 bp)	2	**TGAGT**G**A**, **TGAG**G**C**C
Ver1-b (311 bp)	4	**TTAGTC**C, **TGAG**C**C**T, **TGAGAA**C, **TTAC**GTC
Mcat1-a (259 bp)	1	**TGAGT**TC
Msod (455 bp)	4	**TGAG**G**A**C, **TGAG**GG**A**, **TTACAA**C, **TGAG**GG**A**

1 The nucleotides showing the same sequence with the consensus AP-1 motif (TT/GAC/GT/AA/CA) were indicated in bold.

### MsnA binds promoters of aflatoxin biosynthetic and stress-response genes that carry a STRE motif

**Figure 6:**
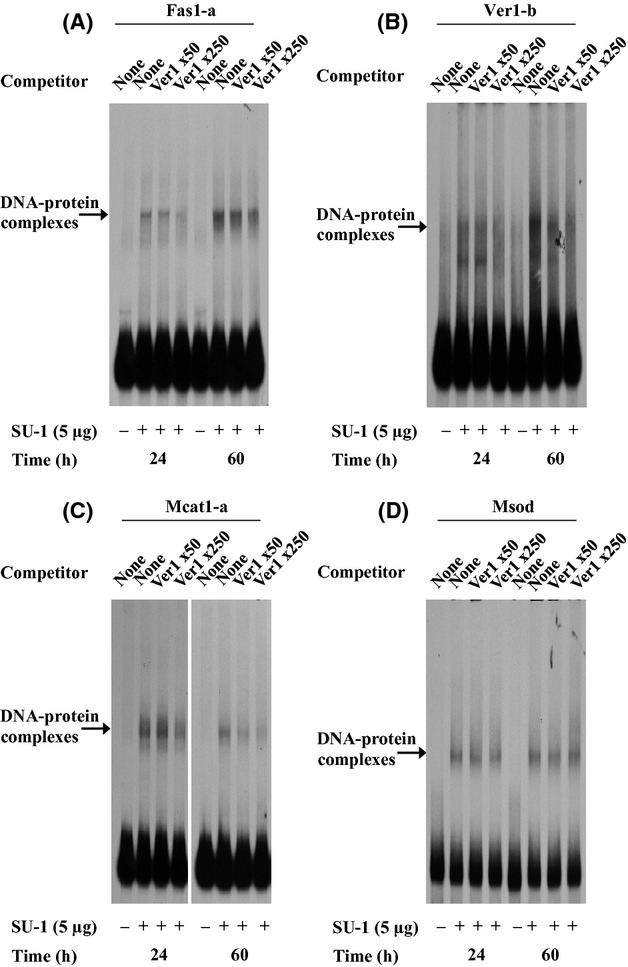
Competition electrophoretic mobility shift assays of *fas-1,* *ver-1*, mycelial *cat1*, and Mn *sod* promoters using a 55-bp Ver1 promoter fragment as a competitor. *Aspergillus parasiticus* SU-1 was grown for 24 or 60 h at 30°C in YES medium. The 55-bp nonlabeled Ver1 promoter fragment (contains both stress-response element and CRE sites; 50- and 250-fold molar excess) was added to compete for labeled Fas1-a, Ver1-b, Mcat1-a, or Msod probes. (A) Fas1-a probe. (B) Ver1-b probe. (C) Mcat1-a probe. (D) Msod probe.

Msn2p and Msn4p (C_2_H_2_-type zinc-finger regulators) bind to a conserved STRE (5′-CCCCT-3′) in promoters of stress-response genes (e.g., cytosolic catalase gene, *CTT1*) in response to oxidative stress (H_2_O_2_) and activate their expression in *S. cerevisiae* (Marchler et al. 1993; Martinez-Pastor et al. 1996). The Msn2p ortholog, MsnA, was identified in *A. parasiticus* and *A*. *flavus* (Chang et al. 2011). Because of the possible link between MsnA and regulation of stress response, we searched for a STRE motif in promoters of aflatoxin and stress-response genes in *A. parasiticus*; we identified a STRE motif in the promoters of *fas-1*, *ver-1*, mycelial *cat1*, and Mn *sod* (Fig. 1). To test whether the conserved STRE motif is involved in DNA–protein complex formation at these promoters, we conducted competition EMSA. We used a 55-bp DNA fragment derived from the *ver-1* promoter which contains both STRE and CRE motifs (5′-CCCCTGGGTCA-3′) (Fig. 1C) to compete for DNA–protein complex formation. The 55-bp Ver1 fragment competed for DNA–protein complex formation in Fas1-a, Ver-1b, and Mcat1-a-promoter fragments using 24- or 60-h nuclear protein extracts (Fig. 6A–C). However, the 55-bp Ver1 promoter fragment did not compete for DNA–protein complex formation in the Msod promoter fragment using the 24- or 60-h nuclear protein extracts (Fig. 6D). To identify complexes dependent on STRE only, competition EMSA was performed using a 55-bp Ver1 m fragment which carries the STRE motif and a mutated version of the CRE motif (TGAC changed to CTTC) (Fig. 1C). EMSA using the 55-bp Ver1 m fragment as a competitor showed the same results as those using the 55-bp Ver1 fragment (Fig. S3). These data suggest that a DNA–protein complex binds the STRE motif in *fas-1*, *ver-1,* and mycelial *cat1* promoters, and this complex contains MsnA. However, MsnA is not part of the DNA–protein complex that forms in the Mn *sod* promoter.

**Figure 7:**
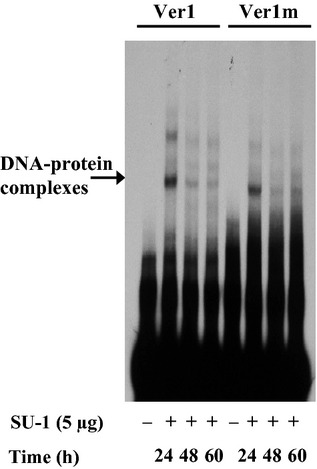
Time-course of DNA–protein complex formation as detected by electrophoretic mobility shift assays using oligonucleotide probes carrying a stress-response element (STRE) site. *Aspergillus parasiticus* SU-1 was grown for 24, 48, or 60 h at 30°C in YES medium. Enriched nuclear protein extracts were prepared as described in Experimental Procedures. Five micrograms of enriched nuclear protein extracts was added to the P^32^-labeled 55-bp Ver1 (contains STRE and CRE sites) or Ver1 m (contains STRE site) oligonucleotide probes in each lane.

To analyze time dependence of DNA–protein complex formation in the 55-bp Ver1 and Ver1 m fragments, EMSA was conducted using 24-, 48-, and 60-h nuclear protein extracts. We observed two major DNA–protein complexes (upper and lower) and minor DNA–protein complexes (Fig. 7). The intensity of the two major DNA–protein complexes at 24 h was maximal and decreased at 48 and 60 h (Fig. 7). The timing of appearance and quantity of the lower DNA–protein complex on the 55-bp Ver1 promoter fragment was similar to that of the Ver1 m fragment, supporting the idea that formation of the lower DNA–protein complex is mediated though the STRE motif. However, formation of the upper DNA–protein complex was dependent on the CRE site. Formation of complexes on both Ver1 and Ver1 m promoter probes was parallel to the expression pattern of mycelial *cat1* described above (Fig. 2B). Interestingly, we observed one minor DNA–protein complex located between the two major DNA–protein complexes. However, it appears the minor complex formation was not affected by the mutation in the Ver1 m fragment.

**Figure 8:**
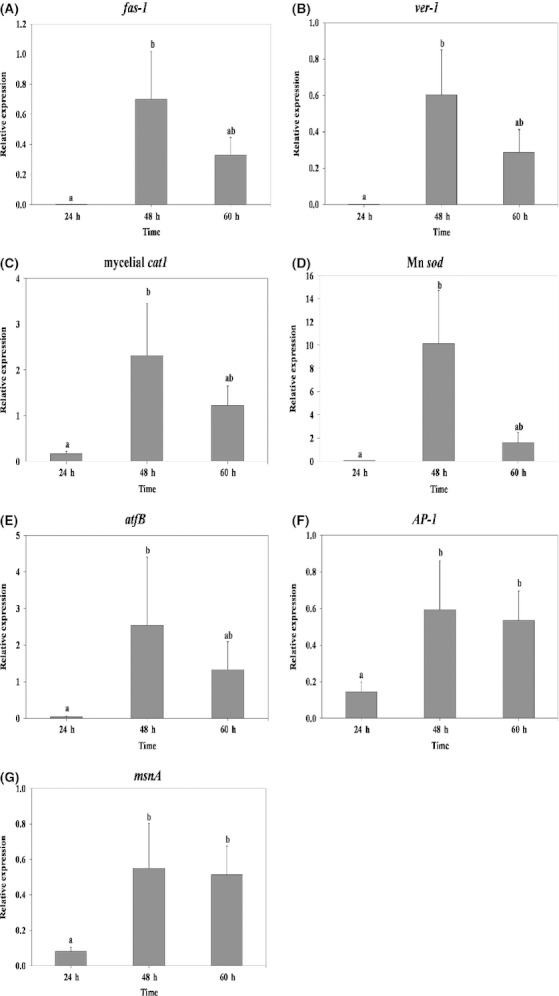
Expression of *fas-1,* *ver-1*, mycelial *cat1*, Mn *sod*, *atfB*, *AP-1*, and *MsnA*. *Aspergillus parasiticus* SU-1 was grown for 24, 48, or 60 h at 30°C in YES medium. Total RNA extraction and real-time PCR analyses were performed as described in Experimental Procedures. The relative level of mRNA is represented as the mRNA level of the target gene divided by the mRNA level of β-tubulin at the same time point. Bars represents mean ± SE (*n* = 4). Statistical analysis was performed by one-way repeated measure analysis of variance. Same lowercase letter indicates that there is no statistically significant difference between two measurements. Different lowercase letters specify a statistically significant difference between two measurements. *P* values are as follows: *fas-1* and *ver-1* (*P* = 0.002), mycelial *cat1* and *atfB* (*P* = 0.011), Mn *sod* (*P* < 0.001), and *AP-1* and *MsnA* (*P* = 0.015). Two independent biological replicates were performed showing the same trend. Two duplicates samples were analyzed for each biological replicate.

These data suggest that MsnA is already bound to promoters of stress-response genes such as mycelial *cat1* at 24 h in order to activate gene expression in response to oxidative stress (H_2_O_2_). However, although MsnA binds to promoters of aflatoxin genes such as *fas-1* and *ver-1* at 24 h, it is unable to activate these genes (Figs. 6, 8). MsnA activation of aflatoxin genes could require activity of additional transcription factors such as AflR and AtfB.

### Expression of genes encoding aflatoxin biosynthetic and antioxidant enzymes and stress-related transcription factors

We analyzed levels of transcripts of early and middle aflatoxin biosynthetic genes (*fas-1* and *ver-1*), antioxidant genes (mycelial *cat1* and Mn *sod*), and oxidative stress-related transcription factor genes (*atfB*, *AP-1*, and *msnA*) in *A. parasiticus* SU-1 grown in aflatoxin-inducing YES medium at 24, 48, or 60 h using real-time PCR. Transcripts of *fas-1, ver-*1, and Mn *sod* were barely detected at 24 h but increased significantly at 48 h and stayed unchanged at 60 h (Fig. 8A, B, and D). This pattern of expression is consistent with the pattern of expression of aflatoxin genes observed previously (Roze et al. 2011). In contrast, the antioxidant gene encoding mycelial *cat1* and genes encoding three oxidative stress-related transcription factors (*atfB*, *AP-1*, and *msnA*) were all expressed at relatively high levels at 24 h and remained unchanged at 48 and 60 h (Fig. 8C and E–G). These data support the EMSA data shown in Figure 2 and indicate two distinct patterns of gene expression. We propose that expression of the aflatoxin genes (*fas-1* and *ver-1*) and Mn *sod* is regulated by a similar mechanism, whereas expression of mycelial *cat1* and the oxidative stress-related transcription factor genes (*atfB*, *AP-1*, and *msnA*) is regulated by a different mechanism.

## Discussion

Our work for the first time provides strong evidence to suggest that at least four transcription factors AtfB, MsnA, SrrA, and AP-1 act together to regulate genes involved in stress response and secondary metabolism. The target genes are located on different chromosomes (based on the *A. flavus* genome). The aflatoxin gene cluster that carries *fas-1* and *ver-1* is located on chromosome 3, the antioxidant gene, mycelial *cat1,* is located on chromosome 5, and Mn *sod* is located on chromosome 2. Nucleotide sequence analysis demonstrated that the promoters of mycelial *cat1* and Mn *sod* in *A. parasiticus* were highly conserved with each other and also showed 90% identity to the corresponding promoter regions in *A. flavus* (Fig. S4). In contrast, the promoters of aflatoxin genes in these two species exhibited significantly lower identity (50%) (Ehrlich et al. 2005). This observation may suggest that the regulation of expression of antioxidant genes is more highly conserved evolutionarily than the regulation of aflatoxin genes. Interestingly, we found one additional CRE site in the promoter of mycelial *cat1* in *A. parasiticus* compared with that in the promoter in *A. flavus*.

The formation of DNA–protein complexes that included AtfB in the aflatoxin and antioxidant promoters correlated strongly with the onset of transcription and the pattern of transcript accumulation in the target genes. These and previous data indicate that AtfB and AflR together function as activators of aflatoxin gene transcription (Roze et al. 2004, 2011) and that the interaction of AtfB with the target promoters depends on the presence of at least one CRE element.

Our data also strongly suggest that homo and/or heterodimerization with other transcription factors (AP-1, SrrA, AtfA, or other bZIP transcription factors) contributes to ability of AtfB to bind the CRE site. Based on the data presented in Table 3, ATF-2 (a homolog of AtfA in *A. nidulans*) forms a heterodimer with Jun (a homolog of Yap1 in *S. cerevisiae*) or it forms a homodimer at CRE sites while ATF-1 (a homolog of AtfB in *A. nidulans*) binds as a homodimer in mammalian cells (Abate et al. 1990; Hai and Curran 1991; Deppmann et al. 2006). On the other hand, Lara-Rojas and coworkers suggested that AtfB might interact with AtfA to regulate common target genes as AtfB in *A. nidulans* is similar to Pcr1 in *S. pombe* and Atf1 forms a heterodimer with Pcr1 in *S. pombe* (Lara-Rojas et al. 2011). It was shown that Atf1 and Pcr1 can form a heterodimer and function as a transcription activator or repressor for chromatin remodeling in *S. pombe* (Davidson et al. 2004). Thus, these data and our previous findings (Roze et al. 2004) strongly suggest that AtfB may interact with AtfA in *A. parasiticus* in response to oxidative stress. To address heterodimer/homodimer formation between AtfB and AtfA, we will analyze the interaction between these two proteins on regulation of aflatoxin and antioxidant genes in future work.

**Table 3:** Comparison of transcription factors which bind to CRE and AP-1 sites in *Aspergillus oryzae, Aspergillus nidulans*, *Saccharomyces cerevisiae*, *Schizosaccharomyces pombe*, and mammalian cells

Transcription factors	*A. oryzae*	*A. nidulans*	*S. cerevisiae*	*S. pombe*	Mammalian cells
Binding to CRE sites	AtfA	AtfA	Sko1(Acr1)	Atf1	ATF-2
Binding to CRE sites	AtfB	NR^1^	NR	NR	ATF-1
Binding to AP-1 sites	NR	NapA	Yap1	Pap1	c-Jun

1 NR represents that it is not reported in literature.

Our EMSA data suggest that Mn *sod* in *A. parasiticus* is activated by AtfB later than mycelial *cat1*, in agreement with findings by others (Kawasaki et al. 1997; Estruch 2000; O'Brien et al. 2004; Gessler et al. 2007). Mn SOD is localized to the mitochondrial matrix and is required for detoxification of superoxide generated in mitochondria during respiration. We also hypothesize that MsnA may assist AtfB binding to the mycelial *cat1* promoter stimulating its early expression. We will analyze this possibility in future studies.

**Figure 9:**
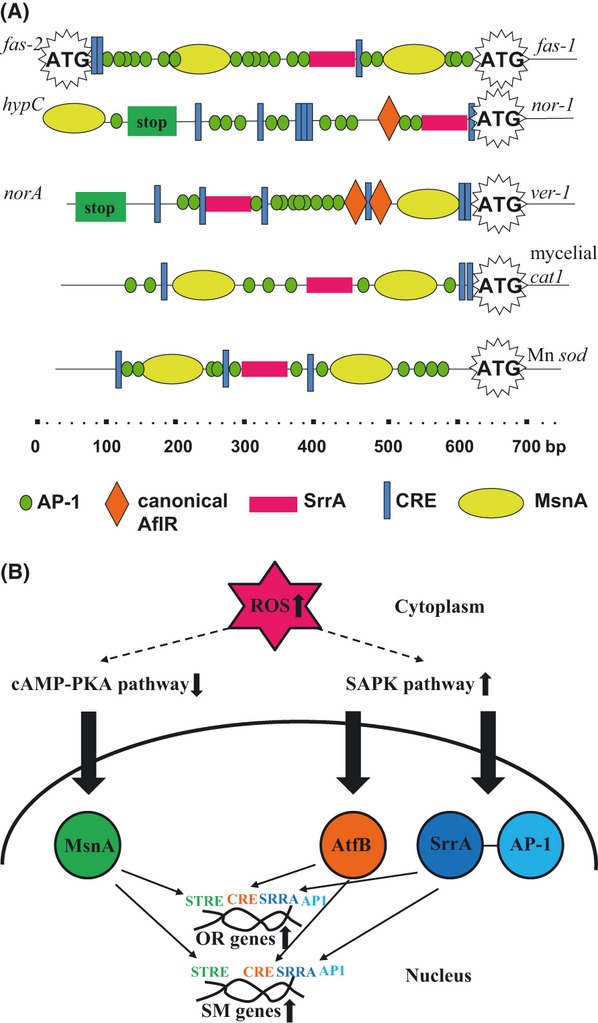
Proposed model for transcriptional activation of secondary metabolism and oxidative stress-response genes by binding of transcription factors in response to oxidative stress. (A) Schematic representation of the transcription factor-binding sites in the promoters of the aflatoxin biosynthetic and antioxidant genes used in this study. (B) Regulatory network for transcriptional activation of secondary metabolism and oxidative stress-response genes for cellular defense against oxidative stress. Based on available experimental evidence, we propose that increased levels of intracellular ROS in fungal cells downregulate the cAMP-PKA signaling pathway. This promotes MsnA binding to stress-response element (STRE) sites in promoters of oxidative stress-response (OR) genes including antioxidant genes for their activation. Simultaneously, ROS upregulates the SAPK signaling cascades. This promotes AtfB and SrrA binding (SrrA recruits AP-1) to corresponding CRE, SRRA, and AP1 sites in promoters of the oxidative stress-response (OR) genes including the antioxidant genes for their induction. Then, MsnA, AtfB, and SrrA bind (SrrA recruits AP-1) to corresponding STRE, CRE, SRRA, and AP1 sites in promoters of secondary metabolism genes including aflatoxin genes for their activation due to excess ROS. Secondary metabolites produced in response to ROS play a role in a defense mechanism of fungal cells against oxidative stress. PKA, protein kinase A; SAPK, stress-activated protein kinase; OR, oxidative stress response; SM, secondary metabolism.

We previously suggested that AP-1 binds at an AP-1 recognition site 5′-TGAGTAC-3′ in the *nor-1* promoter and together with AtfB activates *nor-1* expression (Roze et al. 2011). Although this study does not find AP-1 as part of a stable DNA–protein complex at the promoters of aflatoxin and antioxidant genes, the data do not rule out the possibility that AP-1 is a transient component of complexes at the target promoters. In yeast, the transcription factor Yap1 (Pap1 ortholog) regulates expression of the cytosolic catalase gene *CTT1* (*ctt1*) via association with Skn7 (Prr1 ortholog) in response to H_2_O_2_ (Lee et al. 1999; Nguyen et al. 2000; Toone et al. 2001; Aguirre et al. 2006). Based on this precedent in yeast, *A. parasiticus* AP-1 may function indirectly through a physical association with the ortholog of SrrA (*A. nidulans* ortholog of Skn7 and Prr1 in yeasts) to activate mycelial *cat1* and aflatoxin biosynthetic genes. In support of this model, promoters of the aflatoxin and antioxidant genes (Table 2 and Fig. 9A) contain AP-1-motifs located in close proximity to the SrrA recognition sequence similar to the situation in *S. cerevisiae* (He and Fassler 2005). Alternatively, *A. parasiticus* AP-1 may regulate AflR, a positive regulator of the aflatoxin biosynthetic pathway, in order to activate expression of aflatoxin genes (Reverberi et al. 2010). We will analyze the interaction between AP-1 and SrrA and between AP-1 and AflR on the promoters of the aflatoxin and antioxidant genes in future studies.

The temporal formation of DNA–protein complex in the Mn *sod* promoter differed from that observed in three other promoters analyzed. By what mechanism does *A. parasiticus* regulate the timing of DNA–protein complex formation on different target gene promoters? In theory, the dynamics of DNA–protein complex formation within a specific promoter could be controlled by the landscape of specific *cis*-acting elements in the promoter. In the promoter regions of all five genes analyzed in this study, we observed multiple-binding sites for four transcription factors (AtfB, SrrA, AP-1, and MsnA) located in close proximity to each other and within 500 bp of the ATG translation initiation codon. Each of the five promoter regions contained 3–6 AtfB recognition sites, one SrrA site, one or two MsnA sites, and multiple AP-1 sites. The relative position of these specific *cis*-acting elements was similar but unique in each promoter (Fig. 9A). In three of five promoters regions, CRE sites were detected adjacent to ATG (the exceptions were *fas-1* and Mn *sod*); in four of five promoters analyzed, an MsnA site was detected adjacent (within 100 bp) to ATG (the exception was the *nor-1* promoter).

We previously proposed that AtfB aids AflR binding to its recognition site in the promoter (Roze et al. 2004) and canonical AflR-binding sites 5′-TCGSWNNSCGR-3′ are present in the *nor-1* and *ver-1* promoters (Fig. 9A). Intriguingly, the *A. parasiticus* mycelial *cat1* promoter contained five motifs that differ from the canonical AflR-binding site by only one nucleotide in the 3′ end (TCGAGTCGCCA, TCGCCAGACGG, TCGTAGCCCCA, TCGCAGTGGGA, and TCGTGTTGCCA). The Mn *sod* promoter contained two such motifs (TCGTAGGTGGA and TCGTGCGCCAA), and the *fas-2*/*fas-1* intergenic region possessed one motif TCGAACACCTA. Currently, the functional significance of these motifs is unknown. Further analyses are required to determine the binding specificity of AtfB to these promoters in vitro and in vivo, and also how the landscape of transcription factor recognition sites affects interaction of AtfB with SrrA, AP-1, MsnA, and AflR, its interaction with the promoter, and the transcriptional impact during the response to oxidative stress.

Aflatoxin biosynthesis is triggered as a part of the cellular response to oxidative stress. What is the role of aflatoxin biosynthesis in this response? Does it protect the cell from oxidative stress? In plants and fungi, secondary metabolites play a protective role in adaptation to various biotic and abiotic stressors in the environment. In particular, plants produce many compounds such as phenylamides, polyphenol, and flavonoids including anthocyanin and quercetin, which have ROS-scavenging properties, for their defense (Edreva et al. 2008). Filamentous fungi also produce secondary metabolites with antioxidant function (Aguirre et al. 2006). For example, the addition of resveratrol, a natural antioxidant, to *Aspergillus ochraceus* in culture and catalase to *Fusarium graminearum* in culture reduced synthesis of ochratoxin A and deoxynivalenol, respectively (Fanelli et al. 2004; Ponts et al. 2007). ROS is generated in *A. parasiticus* under aflatoxin-inducing conditions early during growth (8–10 h for O_2_·^−^ generation and 18 h for H_2_O_2_ generation) (Reverberi et al. 2008). Our current data suggest that the expression of mycelial *cat1* is activated by MsnA binding at a STRE site and AtfB binding at CRE sites in the promoter at 24 h. Based on this observation, we propose that antioxidant genes including mycelial *cat1* are activated as the first line of defense against ROS formation, and aflatoxin genes are activated at a later time point as the second line of defense to excessive levels of ROS in agreement with findings presented by Reverberi et al. (2006).

Our data obtained in this study and the observations of others (Toone and Jones 1998; Thevelein and de Winde 1999) enable us to expand our previously proposed regulatory model (Roze et al. 2011) for regulation of stress response and secondary metabolism. We propose that increased levels of intracellular ROS activate antioxidant genes via two signal transduction pathways: First, ROS downregulates the cAMP-PKA pathway, which results in MsnA binding to the target promoter and activation of the antioxidant gene. Simultaneously, ROS upregulates SAPK signaling cascade(s), which results in AtfB and SrrA binding to the promoters and induction of the antioxidant genes. MsnA, AtfB, and SrrA (SrrA recruits AP-1) then bind to the promoters of aflatoxin biosynthetic genes to assist in their induction (Fig. 9B).

In conclusion, in fungi, cellular response to oxidative stress relies on a complex and dynamic network of transcription factors that bind in a highly coordinated manner to the promoters of the target genes and regulate their expression. Understanding the mechanisms that underlie the temporal regulation of a transcription factor binding to its recognition sites in the promoter is one key to establishing the link between intracellular ROS formation, cellular antioxidant defense, and the benefits for survival offered by secondary metabolism.

## Supporting Information

**Figure S1.** Western blot analysis of *A. parasiticus* protein extracts using anti-AP-1 antibodies (peptide 103). Cell proteins were extracted from *A. parasiticus* SU-1 grown in YES at 30°C. Western blot analysis was conducted as described in Experimental Procedures. Total cell protein extracts (90 μg per lane) and enriched nuclear protein extracts (200 μg per lane) were resolved by electrophoresis (12% SDS-PAGE). The PVDF membrane with transferred proteins was developed with anti-AP-1 antibodies (103; 5 μg mL^−1^) as a primary antibody and LI-COR goat anti-rabbit secondary antibody conjugated to the fluorescent tag IRDye 680 (Li-Cor Biosciences, Lincoln, NE). CE, cell protein extracts; EE, enriched nuclear protein extracts.

**Figure S2.** EMSA of AP-1 binding at the *fas-1**,* *ver-1*, mycelial *cat1*, and Mn *sod* promoters. *Aspergillus parasiticus* SU-1 was grown for 24 or 60 h at 30°C in YES medium. Enriched nuclear protein extracts were prepared as described in Experimental Procedures. Five micrograms of enriched nuclear protein extracts were added to P^32^ labeled promoter probes of aflatoxin or antioxidant genes. Anti-AP-1 antibodies (peptide 103) or preimmune serum were added to determine whether these could block protein/DNA interaction. (A) Fas1-a probe. (B) Ver1-b probe. (C) Mcat1-a probe. (D) Msod probe.

**Figure S3.** Competition EMSA of *fas-1*, *ver-1*, mycelial *cat1*, and Mn *sod* promoters using a 55 bp Ver1 m promoter fragment as a competitor. *Aspergillus parasiticus* SU-1 was grown for 24 or 60 h at 30°C in YES medium. The 55 bp non-labeled Ver1 m promoter fragment (contains STRE site; 50 and 250-fold molar excess) was added to compete for labeled Fas1-a, Ver1-b, Mcat1-a, or Msod probes. (A) Fas1-a probe. (B) Ver1-b probe. (C) Mcat1-a probe. (D) Msod probe.

**Figure S4.** Alignment of mycelial *cat1* and Mn *sod* promoter sequences in *A. parasiticus* and *A. flavus*. The ATG start codon in each sequence is shown with a shadow and the CRE sites are underlined. (A) mycelial *cat1* promoter sequences. (B) Mn *sod* promoter sequences.

**Table S1.** Primer sequences used to generate double-stranded DNA fragment probes for EMSA.

**Table S2.** Primer sequences used for qRT-PCR analysis.
