# Supplementary figures and images for: Evidence that a transcription factor regulatory network coordinates oxidative stress response and secondary metabolism in aspergilli
Source: Microbiologyopen. 2013 Jan 1;2(1):144–60. doi: 10.1002/mbo3.63 (PMC3584220; doi:10.1002/mbo3.63)

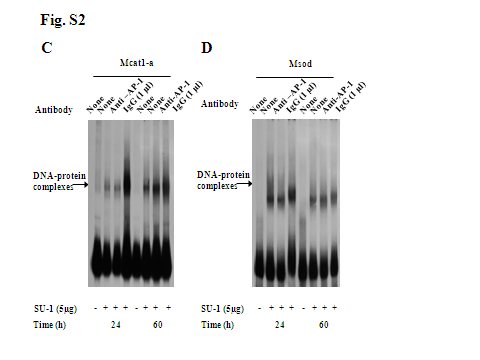


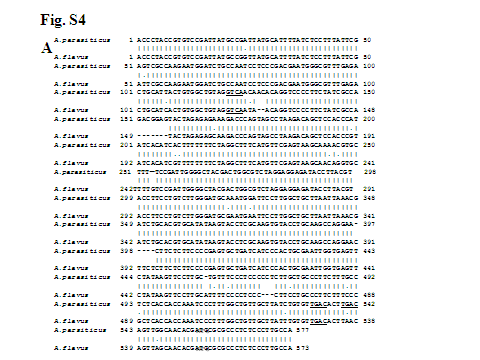

Supplement: Supplementary file 1 [file mbo30002-0144-SD1.docx]
