# Supplementary material for: Evidence that a transcription factor regulatory network coordinates oxidative stress response and secondary metabolism in aspergilli
Source: Microbiologyopen. 2013 Jan 1;2(1):144–60. doi: 10.1002/mbo3.63 (PMC3584220; doi:10.1002/mbo3.63)
Supplement: Supplementary file 2 [file mbo30002-0144-SD2.docx]

**Evidence that a transcription factor regulatory network coordinates oxidative stress response and secondary metabolism in aspergilli**

**Sung-Yong Hong^1^, Ludmila V. Roze^1^, Josephine Wee^1^, and John E. Linz^1,2,3,4*^**

^1^Departments of Food Science and Human Nutrition and ^2^Microbiology and Molecular Genetics,^3^ National Food Safety and Toxicology Center,^4^Center for Integrative Toxicology, Michigan State University, East Lansing, Michigan 48824

*For correspondence. E-mail jlinz@msu.edu; Tel. (+1) 517 355 8474; Fax (+1) 517 353 8963.

**Running title:**

Stress response and aflatoxin biosynthesis

**Keywords:**

aflatoxin/ *Aspergillus parasiticus*/ oxidative stress/ reactive oxygen species/ secondary metabolism

**Supplementary figure legends and tables.**

**Figure S1.** Western blot analysis of *A. parasiticus* protein extracts using anti-AP-1 antibodies (peptide 103). Cell proteins were extracted from *A. parasiticus* SU-1 grown in YES at 30°C. Western blot analysis was conducted as described in materials and methods. Total cell protein extracts (90 µg per lane) and enriched nuclear protein extracts (200 µg per lane) were resolved by electrophoresis (12% SDS-PAGE). The PVDF membrane with transferred proteins was developed with anti-AP-1 antibodies (103; 5 µg ml^-1^) as a primary antibody and LI-COR goat anti-rabbit secondary antibody conjugated to the fluorescent tag IRDye 680 (Li-Cor Biosciences, Lincoln, NE). CE, cell protein extracts; EE, enriched nuclear protein extracts.

**Figure S2.** EMSA of AP-1 binding at the *fas-1, ver-1,* mycelial *cat1*, and Mn *sod* promoters. *A. parasiticus* SU-1 was grown for 24 h or 60 h at 30°C in YES medium. Enriched nuclear protein extracts were prepared as described in materials and methods. 5µg of enriched nuclear protein extracts were added to P^32^ labeled promoter probes of aflatoxin or antioxidant genes. Anti-AP-1 antibodies (peptide 103) or preimmune serum were added to determine whether these could block protein/DNA interaction. A. Fas1-a probe. B. Ver1-b probe. C. Mcat1-a probe. D. Msod probe.

**Figure S3.** Competition EMSA of *fas-1, ver-1,* mycelial *cat1,* and Mn *sod* promoters using a 55 bp Ver1m promoter fragment as a competitor. *A. parasiticus* SU-1 was grown for 24 h or 60 h at 30°C in YES medium. The 55 bp non-labeled Ver1m promoter fragment (contains STRE site; 50 and 250-fold molar excess) was added to compete for labeled Fas1-a, Ver1-b, Mcat1-a, or Msod probes. A. Fas1-a probe. B. Ver1-b probe. C. Mcat1-a probe. D. Msod probe.

**Figure S4.** Alignment of mycelial *cat1* and Mn *sod* promoter sequences in *A. parasiticus* and *A. flavus*. The ATG start codon in each sequence is shown with a shadow and the CRE sites are underlined. A. mycelial *cat1* promoter sequences. B. Mn *sod* promoter sequences.

**Table S1.** Primer sequences used to generate double-stranded DNA fragment probes for EMSA

| DNA Fragment | Sequence^a^ | PCR product (bp) |
| --- | --- | --- |
| *fas-2/fas-1* | F 5’ GCCTCGCACCGCTTTTT 3’  R 5’ CCATCCTTAGACGCAAACA 3’ | 701 |
| Fas2 | F 5’ GCCTCGCACCGCTTTTT 3’  R 5’ AACTATTGGTCGAGCTCCA 3’ | 298 |
| Fas1 | F 5’ ATATATTGATCATCCATGGC 3’  R 5’ CCATCCTTAGACGCAAACA 3’ | 399 |
| Fas1-a | F 5’ TCGCTGAGCTGAAAGGAAGA 3’  R 5’ CCATCCTTAGACGCAAACA 3 | 189 |
| Fas1-b | F 5’ ATATATTGATCATCCATGGC 3’  R 5’ TCTTCCTTTCAGCTCAGCGA 3’ | 230 |
| *norA/ver-1* | F 5’ CCTAGTGGTGGCGGAATATT 3’  R 5’ CTGTCACCAAGGCCACT 3’ | 811 |
| Ver1-a | F 5’ CACGTTAAGAGTATTTTCCAA 3’  R 5’ CTGTCACCAAGGCCACT 3’ | 499 |
| Ver1-b | F 5’ CCTAGTGGTGGCGGAATATT 3’  R 5’AGTATTTCGCTTGTTCGTTTGA 3’ | 311 |
| *omtA/ordA* | F 5’ GTTTATGGGGCTAGGTGT 3’  R 5’ GGTCGGGTGGAAGGCAAG 3’ | 1320 |
| OmtA | F 5’ GTTTATGGGGCTAGGTGT 3’  R 5’ AACGGGCATCTGAAACGG 3’ | 596 |

^a^ F represents forward primers and R represents reverse primers.

**Table S1 (continued).** Primer sequences used to generate double-stranded DNA fragment probes for

| DNA Fragment | Sequence^a^ | PCR product (bp) |
| --- | --- | --- |
| OrdA | F 5’ TTGTCCCTTGATTAATGGTAT 3’  R 5’ GGTCGGGTGGAAGGCAAG 3’ | 692 |
| Vbs | F 5’ GACATATATATCCGCTCGG 3’  R 5’ CATGGCTGTTACCTGGAA 3’ | 298 |
| Mcat1 | F 5’ ACCCTACCGTGTCCGATTATG 3’  R 5’ TGGCAAGGGAGAGGGCGCG 3’ | 577 |
| Mcat1-b | F 5’ ACCCTACCGTGTCCGATTATG 3’  R 5’ AAGCAGCCAAGGAATCCATTT 3 | 339 |
| Mcat1-a | F 5’ AAATGGATTCCTTGGCTGCTT 3’  R 5’ TGGCAAGGGAGAGGGCGCG 3’ | 259 |
| Msod | F 5’ CATAGTATAGTTCTTGTGGG 3’  R 5’ GAGGGAGAATGTGGTGGC 3’ | 455 |
| NorR4 | F 5’ TTTCAACATTTCTTGAGTAC 3’  R 5’ TGATCCGTTCATTATGTCAC 3’ | 51 |
| Fas | F 5’ ATGCTTTAATAGCCGTGAAATAGTAGCG 3’  R 5’ CGCTACTATTTCACGGCTATTAAAGCAT 3’ | 28 |
| Ver1 | F 5’ CGTATATATGTACTACATGCCCGTTCCCCT-  GGGTCACCGTTTTCACAGAACTACA 3’  R5’ TGTAGTTCTGTGAAAACGGTGACCCAGGGG-  AACGGGCATGTAGTACATATATACG 3’ | 55 |

EMSA

^a^ F represents forward primers and R represents reverse primers.

**Table S1 (continued).** Primer sequences used to generate double-stranded DNA fragment probes for

| DNA Fragment | Sequence^a^ | PCR product (bp) |
| --- | --- | --- |
| Ver1m | F 5’ CGTATATATGTACTACATGCCCGTTCCCCT-  GGG AAGCCGTTTTCACAGAACTACA 3’  R 5’ TGTAGTTCTGT GAAAACGGCTTCCCAGGG-  GAACGGGCATGTAGTACATATATACG 3’ | 55 |

EMSA

^a^ F represents forward primers and R represents reverse primers.

**Table S2.** Primer sequences used for qRT-PCR analysis

| Gene | Sequence^a^ |
| --- | --- |
| *fas-1*  *ver-1*  Mycelial *cat1* | F 5’ ATGCATTTGTTCAGCCGACGTGAC 3’  R 5’ TGTCCAATTACCGCCTCCACATCT 3’  F 5’ CGGTGCGCCATTTTGG 3’  R 5’ GGTGACCGAACGATACAATTCC 3’  F 5’ CACGAACGTGTTCCTGAACGT 3’  R 5’ AAGACACCATGGGCACCAA 3’ |
| Mn *sod* | F 5’ GTACTTCAATAACAAGGCCTCGTATG 3’  R 5’ CGCAGTGCGCCAGTTG 3 |
| *atfB* | F 5’ CACCTGCGCAGCGAAGT 3’  R 5’ CGCATGCCGCAGCAT 3’ |
| *msnA*  *AP-1* | F 5’ CCTGTCTTCATCAACGCCAAT 3’  R 5’ GTGGGCAGAGAGGAAGTCTGA 3’  F 5’ GGCCCATTGACCAAGTCAAACCAA 3’  R 5’ CGGTTGTTTGAGCCGTTGAGTGTT 3’ |
| *β-tubulin* | F 5’ AGCTCTCCAACCCCTCTTACG 3’  R 5’ TGAGCTGACCAGGGAAACG 3’ |

^a^ F represents forward primers and R represents reverse primers
